# Assessing the biodiversity and the impact of pollinators on carob production

**DOI:** 10.1371/journal.pone.0291431

**Published:** 2023-10-20

**Authors:** Androulla I. Varnava, George A. Manganaris, Menelaos C. Stavrinides

**Affiliations:** Department of Agricultural Sciences, Biotechnology and Food Science, Cyprus University of Technology, Limassol, Cyprus; Southeastern Louisiana University, UNITED STATES

## Abstract

As the current climate crisis intensifies, drought resistant crops are becoming more important due to their ability to withstand the increasingly hotter and drier summers. Such crops are valuable for pollinators as they provide food resources for wild and managed species. The carob tree (*Ceratonia siliqua* L.) represents an example of a heat- and drought- resistant crop, able to grow in dry areas with practically no inputs. The current study assessed over two growing seasons the diversity of wild bees and other pollinators relying on carob flowers, as well as the contribution of animal pollination to carob production. Carob flowers were subjected to two treatments: Open pollination, where inflorescences were left untreated, and wind pollination, where inflorescences were bagged in a mesh during blooming. Weekly observations during blooming showed that *Apis mellifera* was the most frequent floral visitor followed by wild bees and wasps. Carob flowers were visited by at least 10 different wild bee species. Open-pollinated flowers produced significantly more pods, with the benefit ranging from 4 to 16 times higher production, depending on the region. Open pollination led to pods with greater weight, length and number of seeds compared to pods derived from wind pollination. The results of the current study highlight the importance of animal pollination to carob production, as well as the significance of carob trees to wild bee conservation.

## Introduction

Pollination is one of the most important ecosystem services, with most plant species depending on pollination by animals [1–3]. Flower-visiting animals are responsible for the pollination of more than 70% of the leading global food crops, representing 35% of global production [4]. Important pollinator groups include insects, such as bees, butterflies, moths, flies, wasps, beetles and thrips and vertebrates, such as birds, bats, lizards, and other mammals [2, 5]. The estimated global value of pollination ranges from US$195 billion to US$387 billion annually [6]. Human well-being benefits from pollinators, as their presence ensures crop food security, conserves the diversity of wild plants, maintains the production of honey and other beekeeping products, and supports cultural ecosystem services [2, 7].

Domesticated and wild bees are considered the most important pollinators of wild and cultivated plants, with approximately 20,000 species described worldwide [8, 9]. Managed *Apis* species, such as *A*. *mellifera*, are considered the leading pollination providers [10]. However, the threats faced by honey bees over recent years increased the importance of wild bees as alternative or complementary providers of pollination services [11, 12]. Wild bees contribute to the pollination of many crops, such as coffee, melon, tomato, sunflower, canola, blueberries, apple, and almonds, among many others [9, 13]. According to Maclnnis et al. [14] strawberries pollinated by wild bees were heavier than those pollinated by honeybees. The pollination effectiveness depended on wild bee abundance as floral pollen loads were not affected by species richness. In another study, wild bees were identified as the most important pollinators with strawberry fruit produced having fewer malformations, greater weight and longer shelf-life, resulting in higher commercial value [15]. Similar results have also been reported for other fresh fruits, such as sweet cherry [4]. In addition, a higher diversity and abundance of wild bees resulted in enhanced fruit set in apple orchards [12]. The use of managed solitary bees like *Osmia* can increase fruit set in almond orchards, where a small population ensured effective pollination even though domesticated bees were 10 times more abundant [16, 17].

The carob tree (*Ceratonia siliqua* L) is an underutilized crop with descending trends regarding production volumes, yet it represents an excellent model of a heat- and drought- resistant crop, grown in dry areas with practically no inputs [18, 19]. It is an evergreen dioecious species with some hermaphroditic forms and high longevity [20]. Carob tree domestication in the Mediterranean region dates back to the Roman times and was associated with the introduction of scion grafting in the Mediterranean basin [20, 21]. Work by Baumel et al. [21] points out to multiple origins of domestication of locally selected genotypes, as well as scattered long-distance westward dispersals, along migration routes by Romans, Greeks and Arabs. In the last decades, cultivation spread in areas with a similar climate to the Mediterranean, including California, Arizona, Mexico, Argentina, Australia, India, and South Africa [20]. Carob pods have traditionally been used as feed for livestock [22], and for human nutrition, consisting of dietary fiber, sugars, and a range of bioactive compounds, such as polyphenols and pinitol [20, 23, 24].

Carob cultivation is linked to the culture and traditions of Cyprus; in the past the crop has had substantial importance to the rural economy [18]. Currently, the remaining carob trees (ca. 2000 ha) are a defining feature of the high nature value farmland landscape of Cyprus [25], and form together with olive trees the basic constituents of the habitat type 9320 (Oleo-ceratonion—olive tree and carob tree forests) of Annex I of the Habitats Directive (92/43/EEC).

The carob tree is an entomophilous species, mainly pollinated by flies, bees, and wasps but is also wind-pollinated, depending on the habitat [26, 27]. Diurnal and nocturnal entomophilous pollinators visit carob flowers [26]. However, scarce information exists on the contribution of wild bee species to carob pollination, as well as on the importance of carob flowers to wild bee species. Dafni et al. [26] reported two unidentified wild bee species visiting carob flowers, and a few other studies refer to *Apis mellifera* as a key carob tree pollinator [28–30]. Cyprus hosts 369 bee species [31], but no data exist on their importance to carob pollination. Furthermore, there is no information on the contribution of pollinators to carob production. The current work aimed at assessing the contribution of insects to carob pollination towards yield efficiency and at documenting the diversity of wild bees in carob groves.

## Materials and methods

### Bee presence-absence study

**Fig 1:**
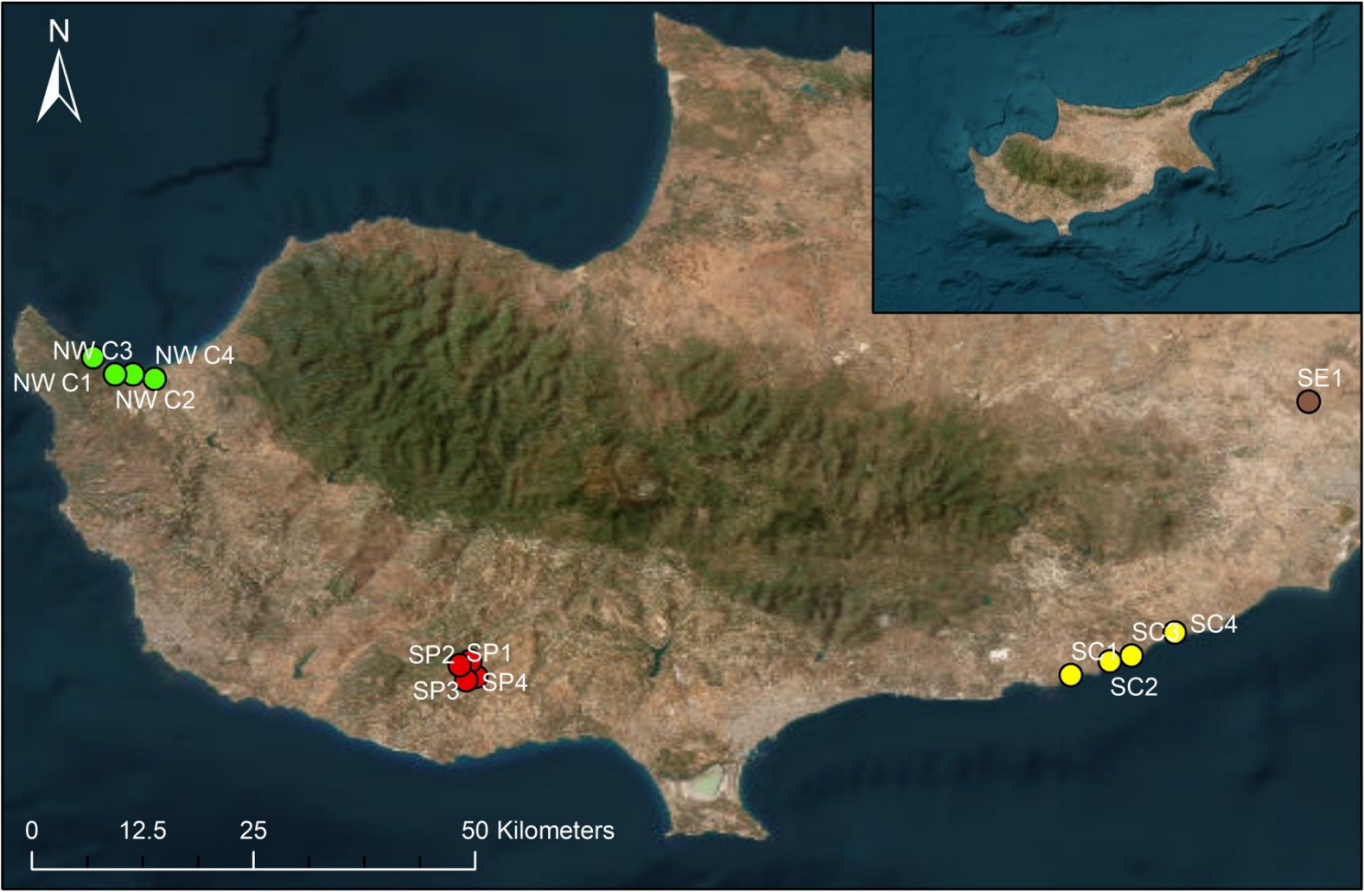
Study sites for the bee presence-absence and carob pollination studies. Bee presence-absence study: North-West Coast–NW C1-4 green dots, South Plateau–SP1-4 red dots, South Coast–SC1-4 yellow dots. South-East Lowlands–SE1 brown dot. ArcGIS v.10.7 [33].

The bee presence-absence study was conducted in the following distinct carob growing regions: (1) North-West Coast (Polis Chrysochous area), (2) the South Plateau (Anogyra area) and (3) the South Coast (Zygi area) in 2016 **(Fig 1)**. NW Coast and the S. Plateau were additionally used for sampling in the 2017 growing season. The three regions represent different landscape types, with the NW Coast classified as settled cultivated coastal lowland, the South Coast as settled cultivated coastal alluvial plain and the South Plateau as settled agrosilvopastoral plateau [32]. Carob groves were located at a minimum distance of 1 km from each other. One male and four female trees were sampled for each of the four carob groves in each region. None of the trees used in the presence-absence study were hermaphroditic. Most carob groves on the island contain a single male tree. Application of pesticides is very uncommon in carob groves, and no pesticide applications were made to the groves selected for the current work.

Each carob grove was sampled weekly for five consecutive weeks during carob blooming on Julian days 294 to 323 in 2016 (October 21 to November 19, 2016) and 293 to 322 in 2017 (October 18 to November 20, 2017). Samplings were conducted between 08:30–12:30 in good weather conditions (sunny days, light breeze, without or slightly cloudy). For each tree, observations were carried out for two min on each of the four cardinal sides of the tree (north, west, south, and east), resulting in a total of eight min per tree and 40 minutes per carob grove (five trees x eight minutes each). For each tree side, data regarding the presence-absence of *A*. *mellifera*, wild bees and other potential pollinators (e.g. wasps, Diptera) were collected. Wild bee specimens representing different morphospecies were collected with a net and transferred to the laboratory for identification at the genus/species level.

### Carob pollination study

The carob pollination study was conducted in three carob groves, one each in the NW Coast (NW C 4), the S. Plateau (SP 1) and the South-East Lowlands (SE 1, **Fig 1**) in 2017. The groves in NW Coast and S. Plateau were selected among the ones used for the bee presence-absence study. The SE Lowlands region is classified as settled lowlands farmland [32]. Based on discussions with growers, the trees in NW C 4 and SP 1 belonged to the landrace Tillyria, while the ones in the SE 1 to the landrace Apostolika, but we note that in many cases it is not possible to separate the two landraces based on morphology alone [34]. None of the landraces used in the pollination study were hermaphroditic.

To assess the contribution of insects to pollination, groups of inflorescences on trees in each carob grove were assigned to two different treatments: a) open pollination, and b) wind pollination. In the open pollination, carob tree inflorescences were not subjected to any treatment, while in the wind pollination inflorescences were enclosed in an insect-proof mesh bag (1 mm size mesh opening) before the start of blooming. Although the insect-proof mesh bags are reducing wind movement and potentially pollen delivery to the flowers, there is no other method for preventing insect access to the flowers. For each of the 20 trees per carob grove, two branches were randomly selected with one group of inflorescences per branch assigned to open pollination and another group assigned to wind pollination (20 trees x 2 branches per tree x 1 group of inflorescences per treatment per branch). Carob inflorescences are found in groups on branches. The number of inflorescences per group varied from five to 30 (median = 15) in the SE Lowlands, four to 16 in the S. Plateau (median = 9), and six to 20 in NW Coast (median = 10).

The mesh bags were removed at the end of November, after the end of blooming. All mature carob pods produced were harvested on Julian day 182 of 2018 (July 1^st^, 2018). Carob pods were transferred to the lab, where we measured the weight, length, and number of seeds produced per pod.

To examine the short-term transfer of pollen by the wind from male to female trees we placed pollen traps on one male (the only male tree present in the carob grove) and five female trees in the SE Lowlands carob grove. Female trees were located at 12, 60, 63 109 and 172 m from the male tree. Pollen traps were constructed using 10 cm diameter and 4.5 cm depth plastic bowls, the base of which was covered with 3 cm height gypsum plaster to limit shaking by the wind. A 9 cm Petri dish, with a filter paper covering its base and filled with 30 ml of 99.5% glycerin was placed within the bowl. The pollen traps were hung on the tree branches at the same height, ca. at 2 m from the ground, with a total of four traps for each of the male and five female trees (24 traps in total). The traps remained in the trees for 24 h (18/11/2017-19/11/2017). After collection of the traps, the glycerin was transferred into 50 ml falcon tubes and stored in the lab at -20°C. To retrieve pollen grains present in the glycerin, falcons were centrifuged at 3500 *g* at 4°C for 20 min. If present, a small part of the pollen pellet was removed, dissolved in 50 μl water, stained with fuchsin and observed under a microscope to confirm the presence or absence of pollen grains. The presence of carob pollen grains was confirmed based on morphology using pictures available in the Pollen Atlas of the Honey Flora of Cyprus [35].

### Statistical analyses

All statistical analyses were carried out in the open-source R language and environment for statistical computing (R Core Team, 2021). Data were curated and plotted using the tidyverse package [36].

The bee presence-absence data were analyzed in a generalized linear mixed-effects model framework using the package lme4 (function glmer) [37], with a log link and a binomial distribution. The response variable was wild bee presence (present or absent). Two analyses were carried out. First an analysis of the 2016 data for all three regions with fixed factors region (NW Coast, S. Plateau, S. Coast), week (5 sampling weeks), tree sex (Male or Female), tree side (N, S, E, W), *A*. *mellifera* presence, and wasp presence (present or absent). Τhe second analysis included the NW Coast–S. Lowlands groves which were sampled both in 2016 and 2017. The fixed factors for the model were region (N. Coast, S. Lowlands), year (2016 or 2017), week (5 sampling weeks), tree sex (Male or Female), tree side (N, S, E, W), *A*. *mellifera* presence and wasp presence (present or absent).

Interactions between the fixed factors were not modelled in either analysis, as their inclusion led to model convergence problems. Tree was included as a random factor for both analyses. Including both plot and tree nested within plot as random factors resulted in a singular model fit, indicating that the random effect structure was too complex to be supported by the data. Model fitting was carried out using the Bobyqa algorithm with the maximum number of iterations set to 2*10^9^. The significance of fixed effects was assessed using *F*-tests, with denominator degrees of freedom obtained by running the model in lme as a linear mixed effects model (package nlme) [38], as discussed by Bolker [39]. Model diagnostics were performed in the DHARMa package using the function simulateResiduals [40]. The function runs tests for correct distribution (KS test), dispersion and residuals, as well as the Levene test for homogeneity of variance. All models fitted the data well.

For the carob pollination study, the data on the number of pods produced in open vs wind pollination were analyzed in a generalized linear mixed effects model framework using the package glmmTMB (function glmmTMB) [41]. Preliminary data analyses showed that the pod number data were over-dispersed, with greater variation than that predicted by the poisson model. The package glmmTMB allows for the modeling of data using a log link, and a negative binomial distribution (family nbinom1, where the variance increases linearly with the mean). As fixed effects we included Treatment (open–wind pollination), carob grove (SE Lowlands 1, NW Coast 4, S. Plateau 1) and their interaction, as well as the number of individual inflorescences per inflorescence group. Tree and branch nested within tree were incorporated in the model as random effects. The significance of fixed effects was assessed using the Anova function of the car package [42], which applies Wald chi-square tests. We point our readers to the ongoing discussion about the use of *p*-values and the different options available to evaluate models at the GLMM FAQ site [39].

The data on pod weight were analyzed in a linear mixed effects framework (function lmer) in the package lme4 [37]. The model included treatment (open–wind pollination), carob grove (SE Lowlands 1 vs NW Coast 4 vs S. Plateau 1) and their interaction as fixed effects. The data from SE Lowlands were not included in the analysis as there were only five pods produced by a total of three carob trees in the wind pollination treatment. Tree, branch nested within tree, and inflorescence group nested within branch within tree were incorporated in the model as random effects. Degrees of freedom for *F*-tests were estimated with Satterthwaite’s approximation as implemented in the ANOVA function of the package lmerTest [43].

Model diagnostics were performed in the DHARMa package [40] using the function simulateResiduals, as outlined in the bee presence-absence study. All models fitted the data well, with the exception of pod weight data where there was a slight departure from variance homogeneity of the simulated residuals, which did not seem to be a major issue after checking the plot of simulated scaled residuals.

## Results

### Bee presence-absence study

**Fig 2:**
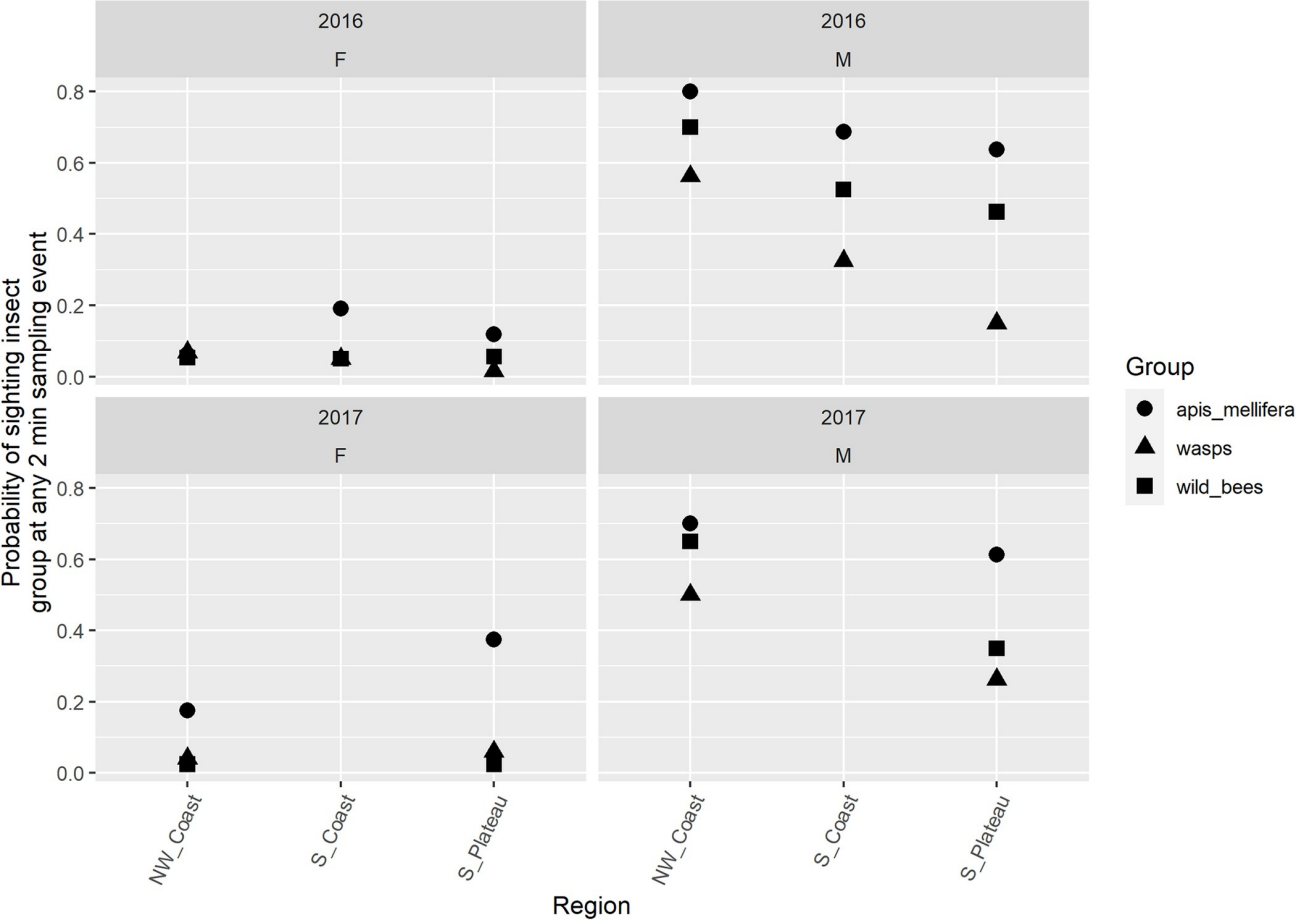
Probability of sighting wild bees, *A*. *mellifera*, or wasps on female (F) and male (M) carob trees in the three sampling regions for 2016 and 2017 at any 2 min sampling event. The S. Coast region was sampled in 2016 only. Each point represents the probability of observing at least one individual of the species group at a sampling point (each of four tree sides, five trees per plot, four plots per region) during five weeks of sampling.

**Fig 3:**
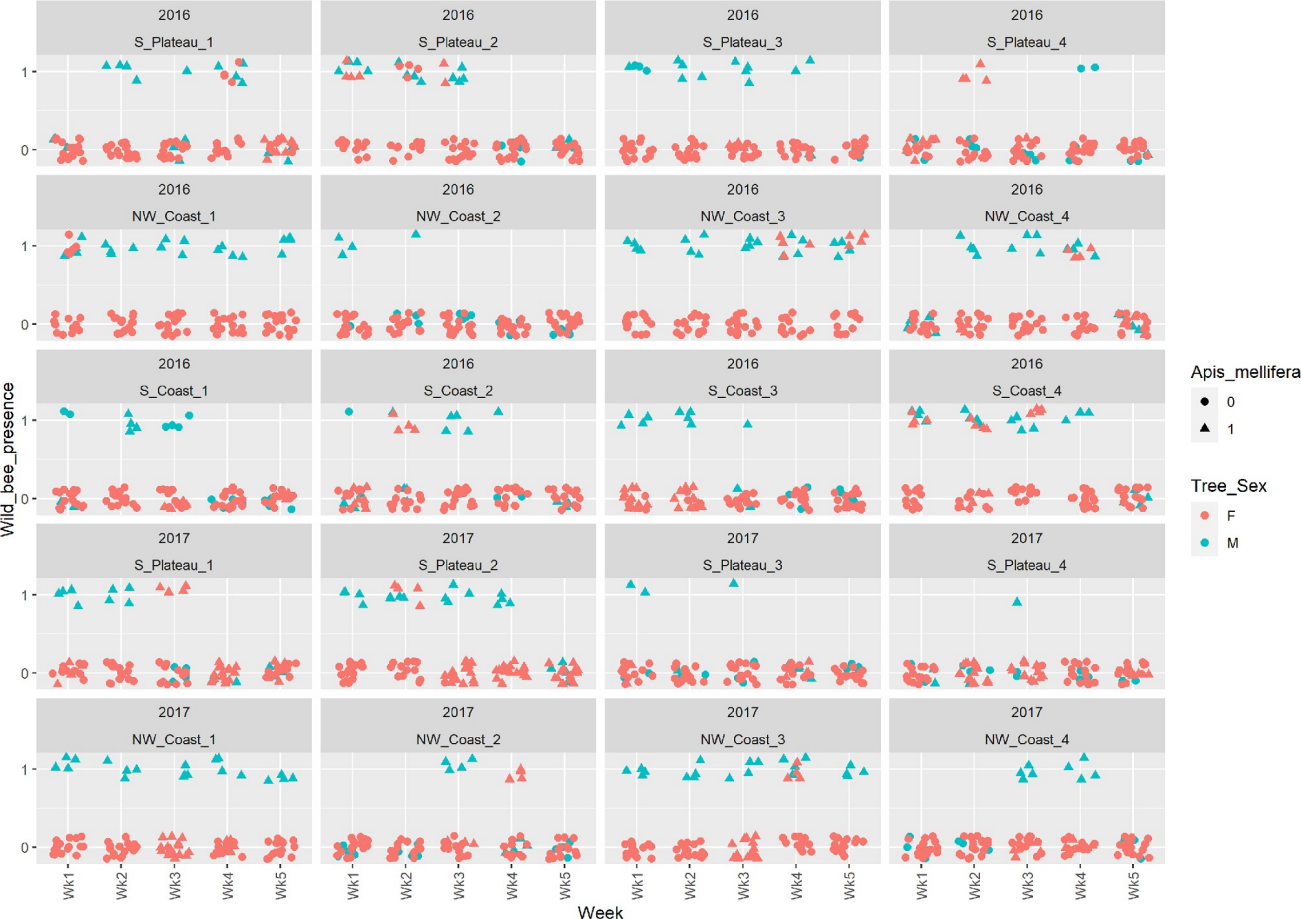
Wild bee presence in the 12 different carob plots sampled for the bee presence-absence study in 2016 and 2017 (S. Coast plots were sampled only in 2016). The graphs show presence or absence of wild bees on four female (red color) and one male tree (cyan) per plot, during five weeks of sampling. All four sites of each tree were sampled, resulting in 20 samples per plot per sampling. Circles show absence of *A*. *mellifera* at the sampling tree site, while triangles show presence of *A*. *mellifera*. Consequently, a circle at y = 1 means that wild bees were present in the absence of *A*. *mellifera*. Horizontal and vertical jitter was added to allow visualization of all data points. See Materials and Methods for more details on sampling.

The overwhelming majority of insect visitors to carob flowers belonged to the order Hymenoptera, i.e. domesticated and wild bees and wasps **(Figs 2 and 3)**, while negligible visits by Lepidoptera or Diptera (Syrphidae) were detected. *Apis mellifera* was the most common species, on both male and female trees, followed by wild bees and wasps **(Fig 2)**. As shown on **Fig 3**, there was a trend of more insect presences at the beginning of carob blooming.

**Fig 4:**
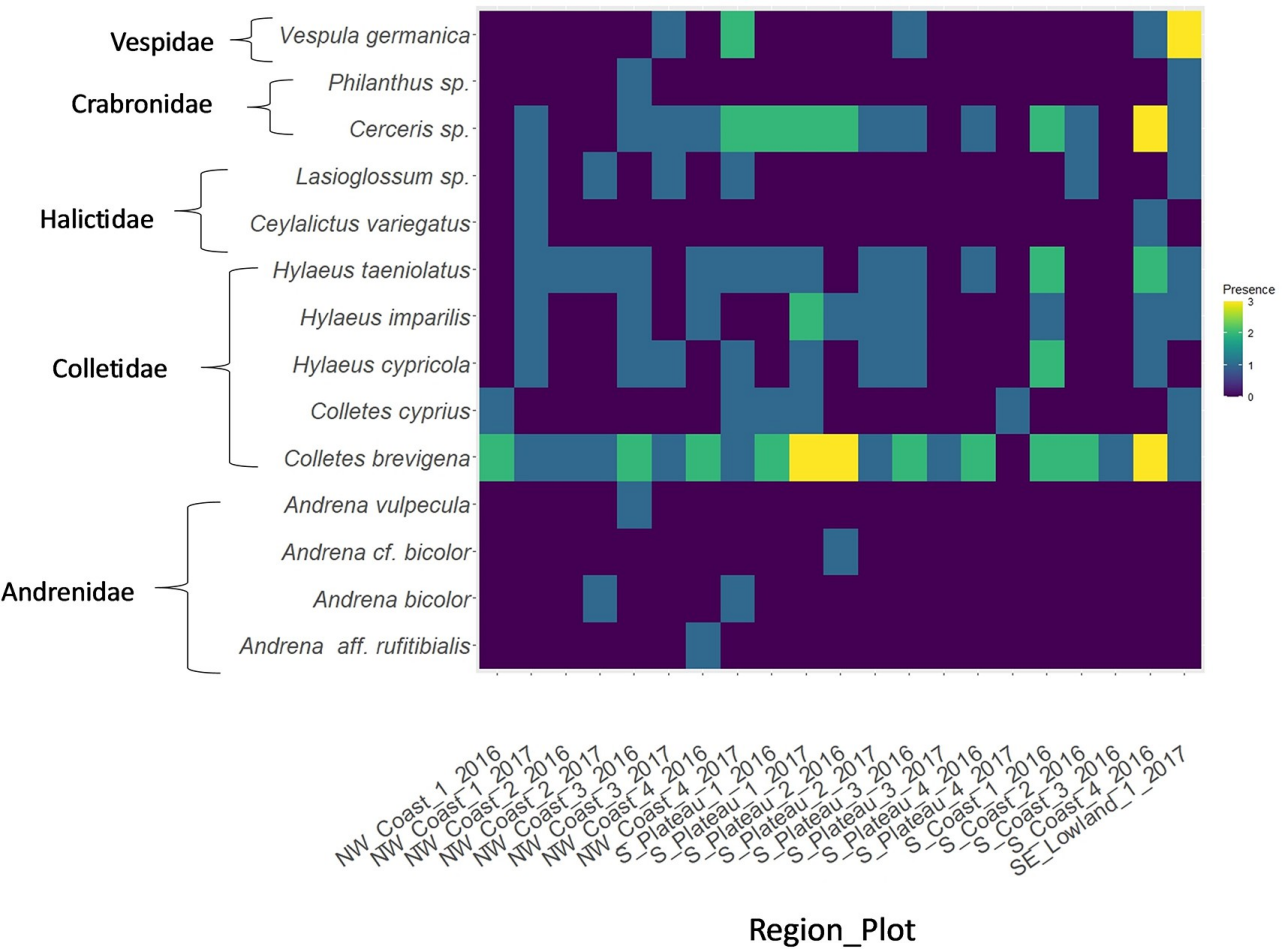
Heat map of wild bee and wasp species collected from carob groves in 2016 and 2017. Each carob grove was visited for five times at weekly intervals during carob blooming each year. The heatmap shows the number of weeks a species was recorded as present in each carob grove.

Identified wild bees belonged to ten different species, representing three families: Halictidae with *Lasioglossum sp*. and *Ceylalictus variegatus*, Andrenidae with *Andrena bicolor*, *Andrena vulpecula* and *Andrena aff*. *rufitibialis* and Colletidae with *Colletes brevigena*, *Colletes cyprius*, *Hylaeus cypricola*, *Hylaeus imparilis* and *Hylaeus taeniolatus*. *Colletes brevigena* and *H*. *taeniolatus* were present in all sampling regions during both years **(Fig 4)**. NW_Coast_4 was the most species-rich plot with eight species represented, followed by NW_Coast_1 with seven species, NW_Coast_3 with six species and S_Plateau_1, S_Plateau_2, S_Coast_4 and SE_Lowland_1 with five species each.

The wasp specimens represented three species belonging to three genera and two families: Crabronidae with *Cerceris sp*. and *Philanthus sp*. and Vespidae with *Vespula germanica* **(Fig 4)**. *Cerceris sp*. and *V*. *germanica* were present in all sampling regions, while *Philanthus sp*. was present only in NW_Coast and SE_Lowland.

The impact of different factors on wild bee presence is shown in **Table 1** (NW Coast, S. Coast and S. Plateau in 2016) and **Table 2** (NW Coast, S. Plateau in 2016 and 2017). In both analyses, there was a significant effect of tree sex, *A*. *mellifera* presence, and wasp presence on wild bee presence (*P* < 0.001). The effect of region, week and year were not significant (*P* > 0.05). Sightings of wild bees were more frequent on male trees and trees with *A*. *mellifera* or wasp presence **(Figs 2 and 3)**.

**Table 1:** Statistical analyses for the generalized mixed-effects model evaluating the effect of region, week, tree sex, tree side, *A*. *mellifera* and wasp presence on wild bee presence in three regions in 2016.

Fixed Effects	df		*F*-value	*P*-value
Region (NW Coast, S. Coast, S. Plateau)	2	56	0.18	0.84
Week	4	1131	1.84	0.12
Tree sex (male or female)	1	56	19.79	< 0.001
Tree side (N, S, E, W)	3	1131	0.48	0.70
*Apis mellifera* presence	1	1131	52.37	< 0.001
Wasp presence	1	1131	27.62	< 0.001
*SD for Random Effect of Carob Tree (*n* = 60)			2.76	
Residual degrees of freedom			1186	

* SD: standard deviation. See Materials and Methods and Results for more information on statistical analyses.

**Table 2:** Statistical analyses for the generalized mixed-effects model evaluating the effect of region, year, week, tree sex, tree side, *A*. *mellifera* or wasp presence on wild bee presence in two regions in 2016 and 2017.

Fixed Effects	df		*F*-value	*P*-value
Region (NW Coast, S. Plateau)	1	37	0.07	0.79
Year	1	1550	0.74	0.39
Week	4	1550	1.95	0.10
Tree sex (male or female)	1	37	22.08	< 0.001
Tree side (N, S, E, W)	3	1550	0.62	0.60
*Apis mellifera* presence	1	1550	59.64	< 0.001
Wasp presence	1	1550	21.83	< 0.001
*SD for Random Effect of Carob Tree (*n* = 40)			2.82	
Residual degrees of freedom			1586	

* SD: standard deviation. See Materials and Methods and Results for more information on statistical analyses.

### Carob pollination study

**Fig 5:**
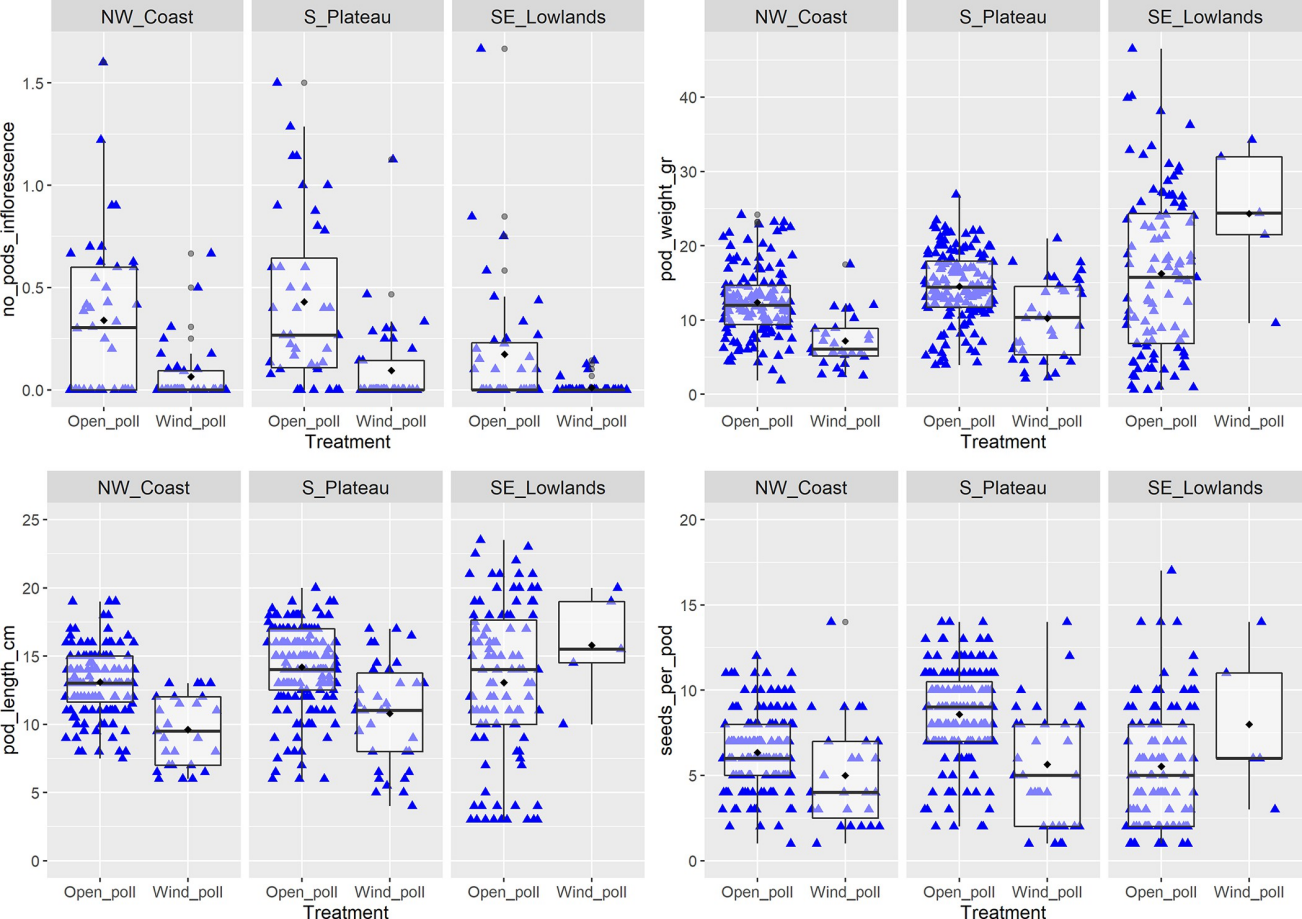
Carob pod number (A), weight (B), length (C), and seeds per pod (D) in the open and wind pollination treatments in the three regions. Boxplots show the median for each treatment, box boundaries show the 25th and 50th percentile, while whiskers extend to 1.5 times the interquartile range (IQR). Points beyond 1.5 times the IQR are plotted individually. Diamonds show the mean. Individual data points are shown with blue triangles. Horizontal jittering was used to allow visualization of all data points.

**Table 3** shows the results of the analysis on the effect of treatment, region and their interaction on the number of pods produced per inflorescence in the open and wind pollination. There was a significant effect of treatment applied (*P* < 0.001) and a weaker effect of region (*P* = 0.02), with no significant interaction between the treatment and the region. The median number of pods per inflorescence was higher in the open than in the wind pollination in all three regions and was higher in the NW Coast followed by the S. Plateau and the SE Lowlands **(Fig 5A)**.

**Table 3:** Results of statistical analyses for the generalized mixed-effects model evaluating the effect of treatment, region and their interaction, and the number of individual inflorescences per inflorescence group on the number of pods produced in the open and wind pollination treatment.

Fixed Effects	df	*χ*^*2*^-value	*p*-value
Treatment (open or wind pollination)	1	52.94	<0.001
Region (NW Coast, S. Plateau, S. Coast)	2	10.13	0.006
Treatment: Region	2	1.13	0.56
No inflorescences per group	1	1.78	0.18
SD* for Random Effect of Tree (*n* = 60)		0.003	
SD for Random Effect of Branch nested within Tree (*n* = 120)		0.67	
Residual degrees of freedom		230	
Dispersion parameter for negative binomial family		3.08	

* SD: standard deviation. See Materials and Methods and Results for more information on statistical analyses.

**Table 4** shows the results of the analysis of the effect of open vs wind pollination and region on carob pod weight **(Fig 5B)**. The median weight per pod was higher in open than in wind pollination with the exception of pods from SE Lowlands in wind pollination, where only five pods were produced from three trees. Median pod weight in open pollination was highest for SE Lowlands, followed by S. Plateau and NW Coast **(Fig 5B)**. A similar trend was observed for pod length, where median length was lowest for pods from NW Coast but the same for S. Plateau and SE Lowlands **(Fig 5C)**, which was highly correlated with pod weight (*r*^*2*^ = 0.74). Pods in the open pollination contained more seeds than those in the wind pollination **(Fig 5D)**.

**Table 4:** Results of statistical analyses for the linear mixed-effects model evaluating the effect of treatment and carob grove on carob pod weight.

Fixed Effects	df		*F*-value	*p*-value
**Treatment** (open vs wind pollination)	1	51.87	28.96	< 0.001
**Carob grove** (NW Coast vs S_Plateau)	1	45.77	6.43	0.01
Treatment: Carob grove interaction	1	51.87	0.32	0.57
SD for Random Effect of Carob Tree (*n* = 38)			1.04	
SD for Random Effect of Branch nested within Carob Tree (*n* = 60)			0.89	
SD for Random Effect of Inflorescence group nested within Branch within Carob Tree (*n* = 78)			2.50	
SD Residual			3.88	

* SD: standard deviation. See Materials and Methods and Results for more information on statistical analyses.

**Fig 6:**
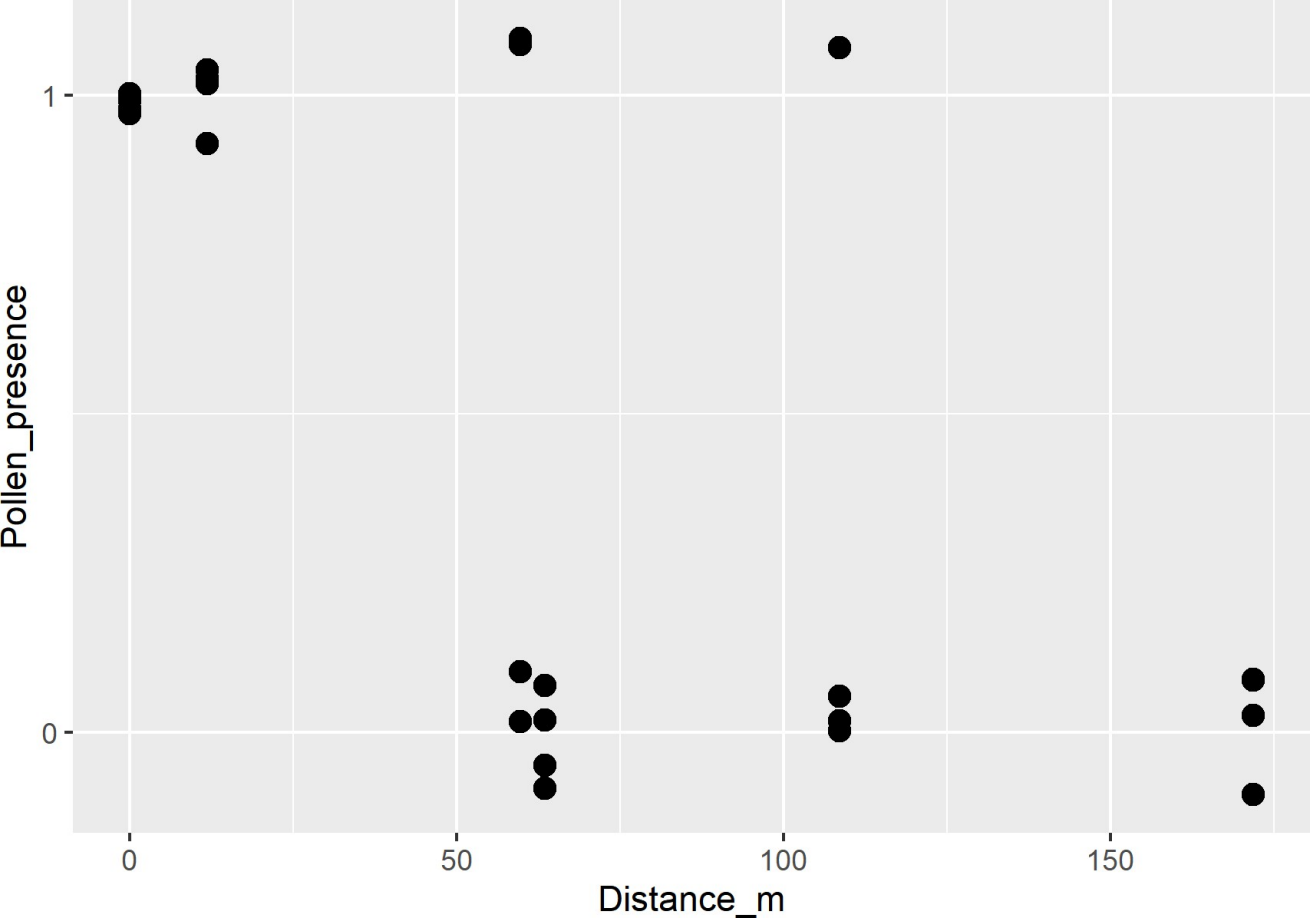
Presence (1) or absence (0) of pollen in pollen traps placed on one male (distance = 0 m) and five female trees. Four pollen traps were placed in each tree for a 24-hour period. Random vertical jitter was added to allow visualization of all data points. See Materials and Methods for more details on the methodology.

All traps placed in the male tree collected pollen **(Fig 6)**, and there was a trend of a lower probability of collecting pollen as the distance from the male tree increased.

## Discussion

The results of the current study highlight the important role of insects in *C*. *siliqua* pollination, fruit set and improved quality of carob pods. Pollination by wind alone resulted in poor yield that was of lower quality. In addition to the most abundant pollinator, the honeybee *Apis mellifera*, we report for the first time five different species of wild bees visiting carob flowers, and potentially providing pollination services. We also note the importance of carob flowers as a feeding source for wild bees, especially during periods of low floral availability.

Bees represented the majority of insects visiting carob flowers during blooming, with *A*. *mellifera* being the most common visitor. Several studies conducted in neighbouring Israel [26], Jordan [45], Spain [30] and Portugal [28] reported *A*. *mellifera* as the most frequent visitor to carob flowers. Even though we did not observe any beehives in the immediate vicinity of the carob groves, hives are distributed in large numbers throughout the island, which explains the high visitation rates by honeybees. Retana et al. [29] found that flies were the most frequent visitors to carob flowers, whereas we recorded negligible visits by Diptera (Syrphidae) and Lepidoptera (butterflies). Although carob flowers are visited by nocturnal insect species belonging to Lepidoptera and Neuroptera [26], the current work assessed only diurnal insect visitors.

Wild bees were the second most frequent visitor to carob flowers, especially on male trees. Only two prior studies reported wild bees visiting carob flowers. *Halictus sp*. and *Amegilla sp*. were recorded from carob flowers in Israel [26] while a companion study by our group reported seven species of wild bees from carob trees in Cyprus: *Lasioglossum anellum*, *Ceylalictus variegatus*, *Colletes brevigena*, *C*. *creticus*, *C*. *cyprius*, *Hylaeus cypricola* and *H*. *imparilis*. *C*. *brevigena* was collected from March to December, *C*. *cyprius* from February to October while *H*. *imparilis* from April to October, with the latter two periods falling outside carob blooming [31]. Five species of wild bees are associated with carob pollination for the first time: *A*. *bicolor*, *A*. *rufitibialis*, *A*. *vulpecula*, *H*. *taeniolatus* and *Lasioglossum sp*.. *C*. *brevigena* seems to rely heavily on carob flowers as well as neighboring habitats, as it was very common in all regions in the current study and was recorded from carob trees in several regions both during and outside the carob blooming period in the past [31].

Wasps were the third most frequent visitor to carob flowers but very few studies refer to them as carob tree pollinators. Dafni et al. [26] recorded very few individuals from two species of wasps, *Vespula germanica* and *Vespa orientalis*, while Arista et al. [44] recorded a high rate of visits (79%) of *Vespula sp*. Al-Ghzawi et al. [45] recorded *V*. *orientalis* and *Polistes dominulus* in small numbers but their contribution to carob tree pollination was small and unreliable.

An earlier study found that male carob trees were more attractive to *A*. *mellifera* and wasp species than female trees, due to pollen production [30], in agreement with the findings of the current work. Visits to flowers of male trees were much more frequent than to flowers of female trees. Male trees are more attractive to insects probably because they provide both pollen and nectar, while female trees provide nectar only, even though female flowers produce a higher volume of nectar with higher sugar content than male flowers [30]. Volatiles emitted by male and female flowers also differ [26]. We note that the visitation rates to male and female trees in the current study are not directly comparable, as in carob groves female trees are found at a much higher density than male trees (ca. 10 female: 1 male). However, there is a strong trend of preference for male versus female flowers by the insect species studied in the current work. The potential impacts of low visitation rates of diurnal species to flowers of female trees may result in pollen limitation. Nocturnal pollinator species carry less pollen than diurnal species [29] and result in lower pollination success [26].

Our finding that carob pollen grains were more likely to be present in pollen traps closer to a male tree suggests that insects are indispensable to carob pollination, as female trees that are distant from male trees might not receive adequate pollen loads from wind alone. However, we note that the pollen transfer study in the current work lasted for 24 h, a very short time period. According to Ortiz et al. [46], the low production of pods is very common in carob trees and does not depend only on the distance from the nearest pollen source but also on blooming phenology.

The open pollination treatment resulted in significantly higher production of pods than wind pollination as described by Dafni et al. [26], with the increase ranging from 4x to 16x higher production. Open-pollinated inflorescences produced pods with a significantly higher weight and length, and higher seed numbers per pod, than wind pollinated flowers. A previous work reported that open-pollinated flowers produced heavier seeds with higher germination rates than bagged flowers [45]. The differences in pod production between the study areas are probably the result of carob landrace / agro-environmental factors as discussed by [34].

Although we did not quantify the exact number of flowers for each inflorescence, our observations at the beginning of flowering suggest that the ca.14 mature flowers per inflorescence reported by Retana et al. [47] in north-east Spain are towards the high end of the numbers we observed in the current work. Retana et al. [47] relying on their own anecdotal observations and past studies report that few *C*. *siliqua* inflorescences produce fruit, and only a small proportion produces more than two fruits, with fruit set at around 5%. In contrast, Ortiz et al. [46] report a mean of almost 41 flowers per inflorescence on carob trees in south Spain, with an average of nine pods per inflorescence, and Arista et al. [44] report a mean of ca. 2.7 pods per inflorescence suggesting that there is high variability within the species and a potential interaction with environmental factors. Pod yield may be influenced by endogenous factors related to alternate bearing, although unfavorable environmental conditions may significantly reduce yield by fruit set reduction [20].

In the current work, each inflorescence produced less than 0.5 pods, which is lower than that reported in previous studies. The low production was similar across regions. In their detailed study Arista et al. [44] showed that even though hand pollination did not increase the number of initiated flowers per inflorescence, it did increase the number of fruits, suggesting an effect of pollen limitation. Yet, even in hand pollinated inflorescences, only a mean of three fruits were produced, suggesting that resources for fruit production are limited. In other words, it seems that fruit production in *C*. *siliqua* is both pollen and resource limited, and in the current study it seems that pollen limitation was an important impediment to production. The impact of pollinators on production could be major, as Arista et al. [44] showed that flowers located towards the apex of the inflorescence have a higher probability of developing into fruits, because pollinators tend to visit the apical flowers first [30, 44].

The positive effect of pollination is not limited to carobs, for instance Nasare et al. [48] recorded heavier shea kernels under open pollination compared with bagged inflorescences. Similarly, tomatoes from non-bagged flowers were larger, heavier, and with more seeds compared with those produced by bagged flowers [49]. Several studies reported that open-pollination can benefit fruit-set, and increase weight and quality [e.g. 11, 12, 50, 51]. Open pollination in kiwifruit resulted in higher fruit set due to insect and especially bee visitation [52]. Several studies on avocado, litchi, longan, guava trees and strawberries highlighted the importance of open pollination to fruit set [53–55].

Farmers are highly dependent on pollinators, reinforcing the fact that pollinator conservation is an anthropogenic pursuit [56, 57]. Beehive renting is common to enhance the pollination of apple trees and other crops [58]. The results of the present study suggest that management to enhance bee populations within or around carob fields could significantly increase pod yield. Wild bees represent an alternative solution, as they inhabit agricultural landscapes and can use pollen and nectar from cultivated crops for their nutrition, simultaneously pollinating the flowers they visit [58]. At the same time, carob trees are important for wild bees. While the current work did not quantify the importance of carob flowers for wild bee nutrition, carob flowers are a very abundant resource during autumn, a period of generally low flower availability in the Mediterranean. Ameliorated understanding of how pollination services can be manipulated could lead to additional practical management guidelines for building sustainability into agricultural systems to protect both, pollinators and crops.

## Conclusions

*Ceratonia siliqua* shows great tolerance to arid and semi-arid climates, an increasingly important trait for environmental, economic, and social reasons because of the changing climate. The current work highlighted the importance of insect pollination and especially of pollinators belonging to the order Hymenoptera, in maintaining high production of carobs, as well as improving pod quality. Taking into consideration the pollination biology of carob trees, further research needs to focus on the identification as well as the assessment of the contribution of night visitors to carob pollination. An additional potential area of future research is the evaluation of the effectiveness of different pollinator species in providing pollination services. Further work on pollen dispersal by wind will improve our understanding of the contribution of wind to carob pollination and can lead to practical recommendations for the density of female vs male trees to maximize production, especially in areas with low pollinator abundance.
